# Simulated Gastrointestinal Digestion of Nutritive Raw Bars: Assessment of Nutrient Bioavailability

**DOI:** 10.3390/foods12122300

**Published:** 2023-06-07

**Authors:** Lucian Dordai, Dorina Simedru, Oana Cadar, Anca Becze

**Affiliations:** National Institute for Research and Development of Optoelectronics INOE 2000, Research Institute for Analytical Instrumentation, 67 Donath Street, 400293 Cluj-Napoca, Romania; lucian.dordai@icia.ro (L.D.); dorina.simedru@icia.ro (D.S.); oana.cadar@icia.ro (O.C.)

**Keywords:** simulated gastrointestinal digestion, raw bars, nutrient bioavailability, digestion, absorption, plant-based diet

## Abstract

Raw bars have become popular among health-conscious consumers due to their nutrient-dense ingredients and lack of additives and preservatives. However, the effect of simulated gastrointestinal digestion on the nutrient content of these bars has yet to be extensively studied. In this study, four different raw bar recipes were subjected to simulated gastrointestinal digestion to evaluate the impact on their nutrient content. The recipes have dates and almond flour as base ingredients and specific ingredients such as Maca root powder, Ginger powder, Aronia powder, Pollen, Propolis extract, Astragalus powder, and Cacao powder. These variations were intended to provide diverse flavors and potential health benefits to cater to different preferences and needs. The in vitro digestion model was designed to mimic the conditions of the human gastrointestinal tract, including the mouth, stomach, and small intestine. The results showed that the simulated gastrointestinal digestion significantly impacted the nutrient content of the bars, with varying degrees of nutrient loss observed depending on the recipe. The highest phenolic content and antioxidant activity were observed in the salivary phase for all samples. Vitamin B content generally decreases from the salivary to the intestinal stage. After digestion, the recovery rates of total phenols, antioxidant capacity, and vitamins B1, B3, and B6 varied across the recipes. The recovery rates of vitamins B1, B3, and B6 were generally high across all recipes, indicating their stability and retention during digestion. The findings suggest that simulated GI digestion provides insights into the nutrient bioavailability of raw bars. These results can inform the formulation and optimization of raw bars to enhance nutrient absorption and nutritional value. Further research is warranted to investigate the effects of different processing techniques and ingredient combinations on nutrient bioavailability.

## 1. Introduction

Raw bars have become increasingly popular as a convenient and healthy snack option, particularly among individuals following a plant-based diet [1]. Over the past five years, snack launches have been dominated by bars in the US, UK, German, and Australian markets [2]. Convenience and the nutritional value of bars are the top reasons for the rising bar consumption [2,3]. To this end, 79% of US consumers have snacked on bars in the past 12 months [4]. A study of UK consumers confirms that bars have reached mass penetration, noting that one-fifth of consumers now eat protein bars at least once a week [5]. This is much more common among young adults, with 54% of those aged 25–34 saying they eat a protein bar once a week or more and 20% saying they eat them daily [6,7]. In addition, one in four consumers eats more protein from plant sources than a year ago [8].

These bars are typically made from whole food ingredients, such as nuts, seeds, and dried fruits, and are sometimes supplemented with additional nutrients [9,10,11]. However, despite their growing popularity, little is known about the digestion and absorption of nutrients from these bars along the human digestive tract [12,13,14]. 

The human digestive tract is a complex system consisting of the mouth, esophagus, stomach, small intestine, large intestine, and rectum [15,16,17]. Each section of the digestive tract plays a specific role in the digestion and absorption of nutrients. For example, the mouth and stomach are responsible for food’s mechanical and chemical breakdown, while the small intestine is the primary site for nutrient absorption [18,19,20].

The nutrient composition of raw bars varies depending on the specific recipe. Still, they are generally high in macronutrients such as carbohydrates, fats, proteins, and micronutrients such as vitamins and minerals [21,22]. However, the bioavailability of these nutrients may differ from that of whole foods due to processing methods and ingredient interactions [23,24].

Understanding the absorption of significant nutrients from raw bars along the human digestive tract is essential for assessing their nutritional value and potential health benefits [25,26]. This information can also inform dietary recommendations and help individuals make informed choices about their food [25].

In this study, our primary objective was to evaluate the simulated GI digestion of raw bars and assess the bioavailability of significant nutrients. Specifically, we will focus on the absorption of macronutrients such as carbohydrates, fats, and proteins and micronutrients such as vitamins and minerals. We will also assess the impact of processing methods and ingredient interactions on nutrient bioavailability.

To accomplish this, a simulated Gastrointestinal Digestion (GID) model was used, which involved simulating the conditions of the human digestive system by subjecting the raw bars to a series of enzymatic and chemical reactions that mimicked the process of digestion [27,28]. 

By investigating the impact of processing methods and ingredient interactions on the digestion and absorption of nutrients, we aimed to provide valuable insights into the nutritional value of raw bars [29]. This information can be utilized to optimize the formulation of raw bars, enhance their nutrient bioavailability, and ultimately improve their efficacy as a plant-based snack option [30].

The outcomes of this study will contribute to a comprehensive understanding of the simulated GI digestion of nutritive raw bars and its implications for nutrient absorption in the human body. Furthermore, this research will assist in expanding our knowledge of digestion processes and nutrient bioavailability, potentially benefiting the development of other functional foods and promoting overall human health. Even though the exact products used may not be commercially available, the recipes can still be used as a starting point for individuals to create their own versions. The focus on raw aspects in this study stems from the growing interest and demand for healthier and plant-based food options. 

## 2. Materials and Methods

### 2.1. Chemicals

All solvents were HPLC grade from VWR (Darmstadt, Germany), and the ultra-pure water was obtained using the ULTRACLEAR UV UF EVOQUA Purification system (Pittsburgh, PA, USA). ACL Kit was from Analitk Jena (Jena, Germany). All other standards used were from Sigma-Aldrich (Saint Louis, MO, USA)

Sodium hydroxide, sodium chloride (NaCl), sodium dihydrogen phosphate (NaH), sodium hydrogen carbonate (NaHCO_3_), potassium chloride (KCl), potassium dihydrogen phosphate (KH_2_PO_4_), calcium chloride dihydrate (CaCl_2_(H_2_O)_2_), magnesium chloride hexahydrate (MgCl_2_(H_2_O)6), ammonium carbonate ((NH_4_)2CO_3_), and hydrochloric acid (HCl) were purchased from Merck (Darmstadt, Germany). α-Amylase from porcine pancreas (enzyme activity 5 U/mg solid), pepsin from porcine gastric mucosa (enzyme activity ≥ 2500 U/mg protein), pancreatin from porcine pancreas (4× USP specifications), and bile bovine were purchased from Sigma-Aldrich (Steinheim, Germany). All chemicals and reagents used were of analytical quality.

All the ingredients for the nutritive raw bars were bought from natural products and supplement shops in Cluj-Napoca, Romania. Table 1 provides information about the ingredients used to prepare the formulations.

Using these ingredients and based on data obtained in other studies, four raw bar recipes have been elaborated. The basic formulation is presented in Table 2.

### 2.2. Sample Extraction for Total Polyphenols, Antioxidant Capacity, and B Vitamins Analysis

#### 2.2.1. Solid Samples

A simplified version of the method applied by Stanikowski et al. was used [31]. A total of 2.5 g of samples were extracted with 10 mL MeOH for 20 min in an ultrasonic bath at room temperature. The samples were centrifuged at 11,000 rpm for 2 min, and the supernatant was filtered through a 0.45 µm cellulose filter. 

#### 2.2.2. Liquid Samples

A total of 1.5 mL of samples was centrifuged at 11,000 rpm for 2 min, and the supernatant was filtered through a 0.45 µm cellulose filter. 

#### 2.2.3. Total Phenolic Compounds Folin–Ciocalteu

A simplified version of the method applied by Stanikowski et al. was used [31]. In total, 5 mL of distilled water, 1.5 mL of sodium carbonate solution (10%), 0.5 mL of sample, and 0.5 mL of Folin–Ciocalteu solution were transferred by pipette into a 15 mL centrifuge vail. After the samples were kept for 45 min at room temperature in the dark, they were measured at a wavelength of 765 nm (Lambda 25, Perkin Elmer, Waltham, MA, USA). The results are expressed in gallic acid equivalent (mg GAE/kg).

#### 2.2.4. Total Antioxidant Capacity

After the methanol extraction, the samples were directly analyzed using the PHOTOCHEM (Analytik Jena, Jena, Germany), and the antioxidant capacity was measured using the ACL kit following the protocol given by the producer. The results are expressed in µg/g or µg/mL equivalent Trolox. 

#### 2.2.5. B Vitamins Analysis

Based on the literature, UHPLC Vanquisher H Dionex (Thermo Fisher Scientific, Germering, Germany) with DAD detector was used for the analysis of the following vitamins: Thiamine (B1), Riboflavin (B2), Nicotinamide (B3), Pyridoxine (B6), and Cyanocobalamin (B12) [32]. Mobile phase was composed of ultra-pure H_2_O with 1% acetic acid and MeOH in gradient with a flow of 0.3 mL/min. The chromatographic column used was an Accucore aQ 100 × 2.1 mm, 2.6 μm, from Thermo Fisher, kept at 25 °C. The injection volume was 8 µL, and the detector was set at 270 nm. The results are expressed in µg/g or µg/mL.

### 2.3. Total Fibers

An adapted method of Zhou et al. was used [33]. Enzymatic digestion was carried out on 10 g of homogenized sample using 100 mL water solution containing 1 mL of alpha-amylase and 1 mL of protease for 30 min at 37 °C. The mixture was then filtered through a Whatman filter paper (Grade 41) to remove any insoluble residues. The residue on the filter paper was washed with 50 mL of acetone, 50 mL of ethanol, and 50 mL of ether to remove any non-fibrous components. The residue was then dried at 105 °C in an oven (MEMMERT UN55, Schwabach, Germany). The ash was obtained by calcinating the dry residue in an electrical furnace (ELIOG, KO 14, Römhild, Germany) at 550 °C for 3 h. The total fiber was calculated as the difference between dried residue and ash. 

### 2.4. Calorie Content

According to the literature, 1 g of finely ground dried sample was measured using the C 200 h auto calorimeter (IKA, Königswinter, Germany) [34]. The caloric values of the samples were expressed in kcal.

### 2.5. Nutritional Parameters Analysis

An adapted method of Zhu et al. was used [35]. Using the Tango (Bruker, Ettlingen, Germany), the sample was measured directly without any extraction. Method parameters are as follows: measurement time: 64 s; resolution: 16 cm^−1^; and scan type: rotating. The results were compared to the Bruker-provided calibration curves for vegetable matrixes.

### 2.6. SEM Analysis

The sample surface was examined using scanning electron microscopy (VEGA3 SBU, Tescan, Brno-Kohoutovice, Czech Republic). Furthermore, 3 mm specimens of each sample were used for this study. The magnification levels were between 130–250×, the high voltage (HV) was set at 7 kV, and the working distance at 15 mm. 

### 2.7. Simulated In Vitro Gastrointestinal Digestion of Nutritive Raw Bars

The in vitro gastrointestinal digestion used involved three steps (oral, gastric, and intestinal) and was adapted from Minekus et al. and Brodkorb et al. [36,37]. Briefly, the steps for the in vitro digestion protocol consist of the following: (i) oral phase, i.e., 5 g of homogenized sample is mixed with simulated salivary fluid (SSF) (1:1) and amylase (75 U/mL) for 2 min at pH = 7; (ii) gastric phase, i.e., the oral bolus is mixed with simulated gastric fluid (SGF) (1:1) and pepsin (2000 U/mL) and gastric lipase (60 U/mL) for 2 h at pH = 3; (iii) intestinal phase, i.e., the gastric chyme is mixed with simulated intestinal fluid (SIF) (1:1) and pancreatin (trypsin activity 100 U/mL) and bile (10 mM) for 2 h at pH = 7. The solutions were incubated using a Laboshake incubator (Gerhardt, Wiesbaden, Germany). At the end of gastrointestinal digestion, the samples were placed in an ice bath to deactivate the enzymes for 10 min and centrifuged at 4500 rpm for 30 min using a Universal 320 centrifuge (Hettich, Tuttlingen, Germany). The aliquots of supernatants (5 mL) were collected, filtered through 0.45 µm, and stored at 4 °C. A blank sample was incubated under the same conditions and used to determine the phenolic content, antioxidant activity, and B vitamins content in order to correct the interferences from the digestive enzymes and buffers. The analyses were performed in triplicate. 

### 2.8. Statistical Analyses

All of the samples were analyzed three times to ensure the reliability of the results. Statistical analyses were conducted using Minitab Program for Windows version 17.0 (Minitab, LLC, State College, PA, USA), and the data were presented as mean ± standard deviation (SD) by employing the ANOVA technique. To investigate the relationship between the parameters, the Pearson correlation matrix and r² coefficient were used. The differences were considered statistically significant at a significance level of *p* < 0.05.

## 3. Results and Discussion

The four raw bar recipes developed using dates, almond flour, and other ingredients have been evaluated for their nutritional value. The nutritional data of raw bars are presented in Table 3.

According to the data presented, Recipe 1 has the highest total sugar content at 51.11%, followed by Recipe 2 at 44.98%, Recipe 3 at 45.40%, and Recipe 4 at 47.14%. Regarding protein content, Recipe 1 has the highest at 12.13%, followed by Recipe 2 at 9.99%, Recipe 3 at 9.72%, and Recipe 4 at 9.83%. The total lipids content is relatively stable across all four recipes, with Recipe 2 having the highest range at 19.37%, followed by Recipe 1 at 18.77%, Recipe 3 at 18.56%, and Recipe 4 at 18.26%. The total fiber content is highest in Recipes 3 and 2 at 13.86% and 11.86%, respectively, while Recipes 1 and 4 have lower total fiber content at 6.67% and 6.43%, respectively. The calorie content is highest in Recipe 3 at 461.11 kcal and lowest in Recipe 4 at 425.87 kcal. The nutritional composition of the raw bars is generally consistent with the literature on similar products. A study conducted by Silva Lima et al. found that nutritive bars with almonds showed higher protein content (24.95 g/100 g) than Brazil nuts (14.74 g/100 g), while Brazil nuts showed higher lipidic content (59.36 g/100 g), consistent with the data presented here [38]. The lipids and protein content are higher than those found by Parn et al., who found levels of proteins between 3.70–4.06% and lipids between 4.6–5.51% [39]. This is due to the fact that they developed bars that have dates as a main ingredient and no nuts. Another study by Powell et al. found that protein content in nutritive bars ranged from 9–22%, which is also consistent with the protein content in the nutritive raw bars developed in this study [40]. Regarding the specific ingredients used, propolis extract has antimicrobial, anti-inflammatory, and antioxidant properties, which may contribute to the overall health benefits of the raw bars [41,42]. Similarly, maca root powder has been found to have antioxidant and anti-inflammatory effects and potential benefits for energy and immune function [43,44].

The data presented in Table 4 shows the amount of total phenolic content, Trolox equivalent antioxidant capacity (TEAC), and the levels of vitamins B1, B3, and B6 in four different recipes. The table shows each recipe’s mean (±standard deviation SD) across various parameters. The *p*-values also show the statistical significance of recipe parameter differences. Lower *p*-values indicate statistical significance; for all parameters except B6, the *p*-value is much lower than the significance level of 0.05. For B6, the *p*-value is above 0.05, at 0.5628; this proves that the null hypothesis can not be denied, so there is not a significant difference between the recipes. 

In terms of total phenolic content, Recipe 1 has the highest amount at 926.32 mg GAE/kg, followed by Recipe 4 at 844.23 mg GAE/kg, Recipe 2 at 749.1 mg GAE/kg, and Recipe 3 at 489.45 mg GAE/kg (*p* = 2.0206 × 10^−9^). The high total phenolic content in Recipes 1 and 4 suggests they may have more significant antioxidant potential than other recipes. Silva Lima et al. found similar levels of phenolic content ranging from 876 to 943 mg GAE/100 g [38]. 

The TEAC values in all four recipes are relatively consistent, indicating similar antioxidant capacity. This is also consistent with previous studies that have found TEAC values to be a reliable indicator of antioxidant capacity in food [45,46]. Regarding vitamin B1 content, Recipe 1 has the highest amount, followed by Recipe 2, Recipe 4, and Recipe 3. This is consistent with the fact that dates, a significant ingredient in Recipe 1, are a good source of vitamin B1 [47]. The levels of vitamin B3 are highest in Recipe 1, followed by Recipe 4, Recipe 2, and Recipe 3. This is consistent with previous studies that have found dates to be a good source of vitamin B3 [47,48]. The levels of vitamin B6 are highest in Recipe 1, followed by Recipe 2, Recipe 3, and Recipe 4. This is consistent with the fact that dates are a good source of B vitamins [47].

**Figure 1:**
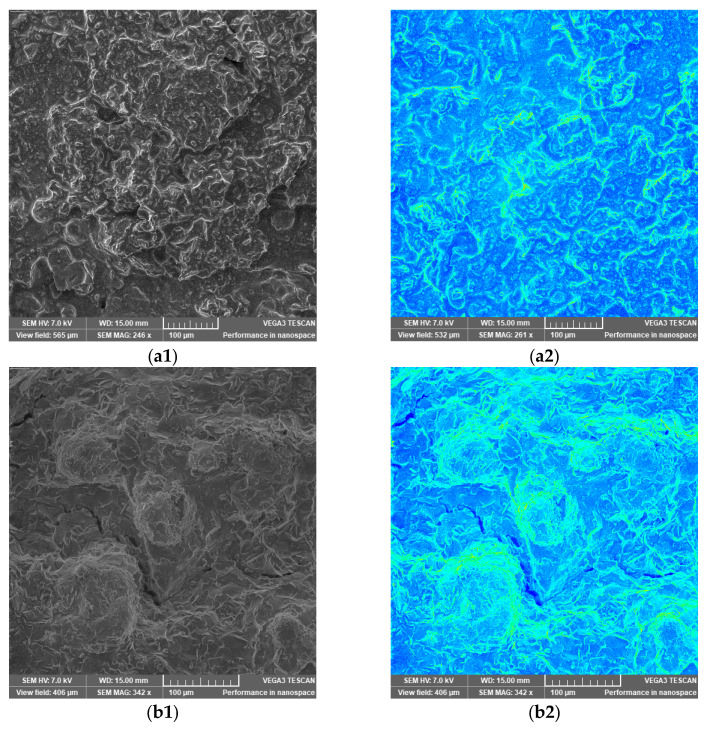

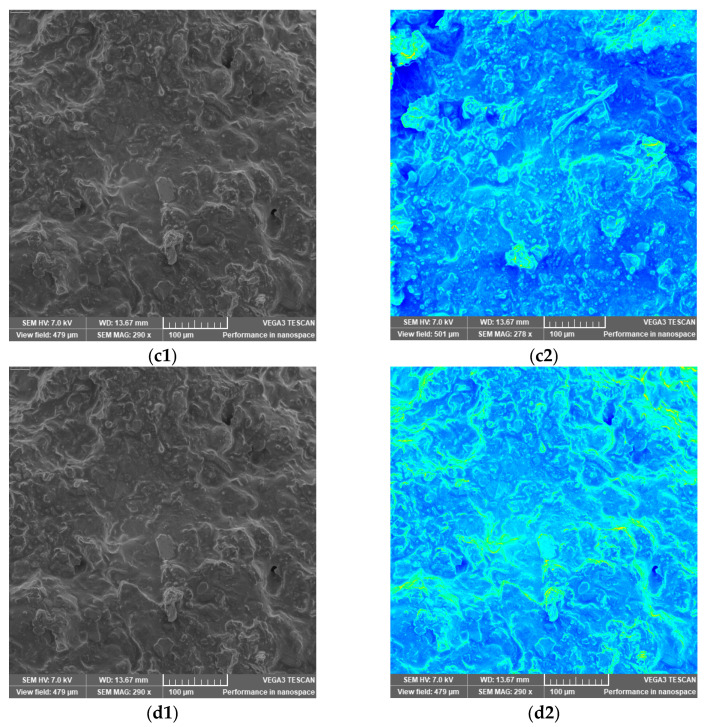
SEM images of Recipe 1 (**a1** without colorize feature, **a2** with colorize feature), 2 (**b1** without colorize feature, **b2** with colorize feature), 3 (**c1** without colorize feature, **c2** with colorize feature), and 4 (**d1** without colorize feature, **d2** with colorize feature).

SEM images of investigated samples are presented in Figure 1. By using SEM’s colorize feature (blue and green colors), the elements on the surface of the bars can be further contrasted so that they are easier to distinguish from each other. Blue covers most sample surfaces, with green lines underlining bumps, suggesting smooth, compact, and continuous surfaces with a homogenous distribution of the ingredients for all bars. Comparing the SEM images, it can be observed that bars 3 and 4 have rougher and more irregular structures. This may be related to the higher amounts of Aronia powder (bar 3) and ginger and asparagus powder (bar 4) with higher particles than the other ingredients present in bars 1 and 2.

In this study, SEM proved to be a useful technique to investigate the homogeneity of the bars’ structures, as it was also suggested in the investigations of several other similar studies [33,49,50]

The data presented in Table 5 describes an in vitro gastrointestinal digestion protocol used to assess the release of phenolic compounds and vitamins B1, B3, and B6 from different food samples during simulated digestion. Compared to other digestibility models, the distinctive aspects of this method lie in the specific parameters used for each phase of digestion, such as the enzymes, pH levels, and incubation times. Additionally, the adaptation from Minekus et al. and Brodkorb et al. [36,37] allows for consistency and comparability with established protocols.

**Figure 2:**
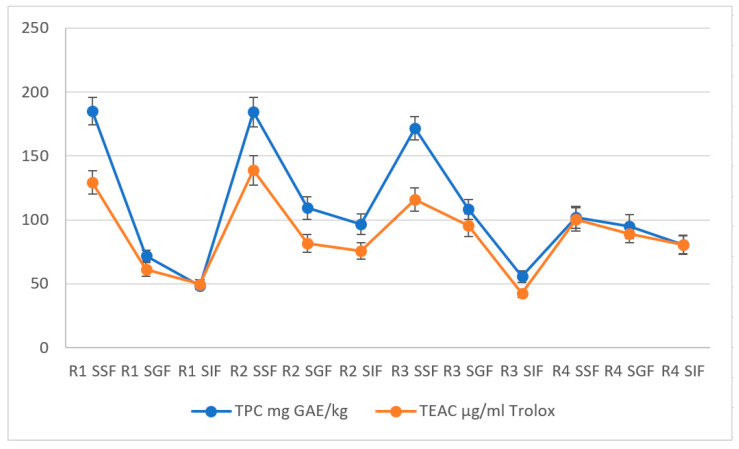
Distribution of total phenols and antioxidant capacity in different digestive fluids (error bars ± SD).

**Figure 3:**
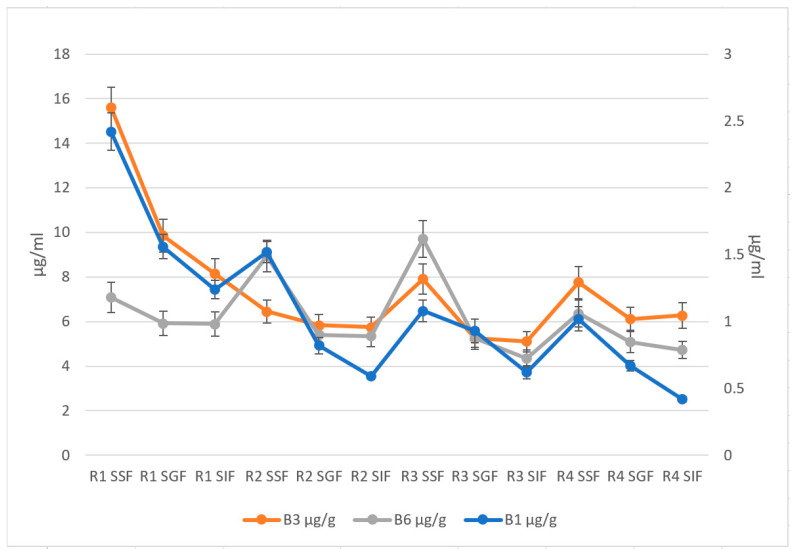
Distribution of vitamins B1, B3, and B6 and antioxidant capacity in different digestive fluids (error bars ± SD, B3, B6 primary axe and B1 secondary axe).

The results indicate that the release of phenolic compounds and vitamins B1, B3, and B6 varied depending on the sample and the stage of digestion (Figure 2). Recipe 1 showed the highest release of phenolic compounds in the simulated saliva phase, while Recipe 2 showed the highest release in the simulated intestinal fluid phase. This is consistent with previous studies that have found that the bioavailability of phenolic compounds is influenced by the stage of digestion and the food matrix [36,51]. Regarding vitamin B1, Recipe 1 showed the highest release in the simulated gastric fluid phase, while Recipe 2 showed the highest release in the simulated intestinal liquid phase (Figure 3). This is consistent with previous studies that have found that the release of vitamin B1 during digestion is influenced by the food matrix and the pH of the digestive juices [52,53]. 

The highest phenolic content is observed in the salivary phase for R1 and R2 samples, while R3 and R4 show the highest salivary degree but similar values between salivary and gastric stages. All models have the most increased antioxidant activity in the salivary phase. The antioxidant activity also depresses from the salivary to the intestinal phase in most cases. The content of B vitamins also generally decreases from the salivary to the intestinal stage, but the pattern is less clear than for the other two parameters. The content of vitamin B1 is highest in the salivary phase for R1, R2, and R4 but most elevated in the gastric phase for R3. Similarly, the content of vitamins B3 and B6 is highest in the salivary phase for R1 and R2 but not for R3 and R4. The findings align with those reported by Catak et al., wherein the in vitro bioaccessibility ranged from 70.9% to 90.2% for vitamin B1, 54.2% to 89.7% for vitamin B2, 42.1% to 94.9% for vitamin B3, and 44.1% to 92.5% for vitamin B6 [54].

Table 6 presents the overall recovery rates (in percentage) of total phenols, antioxidant capacity, and vitamins B1, B3, and B6 for the four recipes after the digestive process.

The recovery of total phenols ranges from 32.9% (Recipe 1 and Recipe 4) to 68.6% (Recipe 3). This suggests that Recipe 3 retains the most phenolic compounds during digestion. Similarly, the antioxidant capacity is highest for Recipe 3 (18.3%) and lowest for Recipe 1 (9.2%). Results are similar to the findings of Linghong Liang et al. that the bioaccessibility of anthocyanins reported was substantially lower following intestinal digestion, with recovery rates of only 0.34% for the IN sample and 4.58% for the OUT sample [55].

The recovery of vitamins B1, B3, and B6 is generally high across all recipes, usually exceeding 90%. This suggests that these vitamins are relatively stable and well-retained during digestion. The results are in the same range as those found by Catak et al., for which the in vitro bioaccessibility was 70.9–90.2%, 54.2–89.7%, 42.1–94.9%, and 44.1–92.5% for vitamins B1, B2, B3, and B6 [54].

Pearson correlation matrix (Table 7) provides values for the linear relationship between pairs of variables analyzed in the study. The deeper the green color is the higher the correlation between parameters, while the more yellow tone green represents a lower correlation. 

The correlation coefficient 0.950391 suggests a solid positive relationship between total phenols (mg GAE/L) and antioxidant capacity (µg/mL equivalent Trolox). Total phenols show a moderate to strong positive correlation with these vitamins, with the strongest correlation seen with B6 (0.80458) and average with B1 (0.565792) and B3 (0.422962). Antioxidant capacity shows a moderate to strong positive correlation with these vitamins, similar to total phenols, with the strongest correlation seen with B6 (0.733938) and moderate with B1 (0.493018) and B3 (0.356062). The strongest correlation among vitamins is between B1 and B3 (0.884969), suggesting a strong positive relationship. The correlation between B1 and B6 is also moderately strong (0.520618). The correlation between B3 and B6 is weaker (0.334375), indicating a less significant linear relationship.

These results suggest that the phenolic content and antioxidant activity are highly correlated, which is to be expected given that phenols are known to contribute to antioxidant activity. The results are similar to the ones found by Hernández-Maldonado et al., which concluded that the mango-based bars that they studied contained high concentrations of polyphenols content, which was in agreement with their high antioxidant capacity [56]. Vitamins B1, B3, and B6 also show moderate to strong correlations with both phenolic content and antioxidant activity, suggesting they may also contribute to these properties.

## 4. Conclusions

The study assesses the nutritional value of the four raw bar recipes developed using dates, almond flour, and various other ingredients, revealing valuable findings. Regarding the dietary components, Recipe 1 outperforms the others in total phenolic content, suggesting a higher potential for antioxidant activity. However, all four recipes show fairly consistent Trolox equivalent antioxidant capacity (TEAC) values, indicating a similar antioxidant potential. The content of Vitamins B1, B3, and B6 was also found to be highest in Recipe 1. The simulated in vitro gastrointestinal digestion study exhibited that the release of phenolic compounds and Vitamins B1, B3, and B6 during digestion varies with the sample and digestion stage. Notably, the highest phenolic content and antioxidant activity were observed in the salivary phase for all samples. Vitamin B content generally decreased from the salivary to the intestinal degree. After digestion, the recovery rates of total phenols, antioxidant capacity, and vitamins B1, B3, and B6 varied across the recipes. Recipe 3 showed the highest retention of phenolic compounds and antioxidant capacity during digestion. The recovery rates of vitamins B1, B3, and B6 were generally high across all recipes, indicating their stability and retention during digestion. These findings provide comprehensive insights into raw bar recipes’ nutritional value and potential health benefits. They also demonstrate the impact of the digestion process on the release and recovery of certain nutrients. Further research is warranted to validate these findings in vivo and to explore the potential health implications of consuming these raw bars. 

## Figures and Tables

**Table 1:** The ingredients of the raw bars.

Name of Ingredients	Description
Dates	Country of origin Iran, 500 g package
Almond flour	Country of origin USA, 250 g package
Maca root powder	100% *Lepidium meyenii* root fine powder. Country of origin Peru, 200 g package
Ginger powder	100% *Zingiber officinale* root fine powder. Country of origin China, 200 g package
Aronia powder	100% organic powder obtained from aronia fruits (shell fragments, pulp, and seeds) dried and finely ground. Country of origin Romania, 200 g package
Pollen	100% raw polyfloral pollen. Country of origin Romania, 100 g package
Propolis extract	Hydroglyceric propolis extract, with a minim of 12 mg/mL polyphenols content (60%), vegetal glycerin 20%, and purified water 20%. Country of origin Romania, 20 mL package
Astragalus powder	100% *Astragalus membranaceus* root fine powder. Country of origin China
Cacao powder	Cacao powder with a minimum of 20% cacao butter. Country of origin non-UE, 200 g package

**Table 2:** The producing recipe.

Name of Ingredients	Recipe 1 (%) R1	Recipe 2 (%) R2	Recipe 3 (%) R3	Recipe 4 (%) R4
Dates	48.6	48.6	48.6	48.6
Almond flour	34.7	34.7	34.7	34.7
Maca root powder	0.7	0	4.2	0
Ginger powder	0	2	0	5
Aronia powder	0	10	12.5	0
Pollen	15	0	0	5
Propolis extract	0	0	0	5
Astragalus powder	0	0	0	1.7
Cacao powder	1	4.7	0	0

**Table 3:** Nutritional data of nutritive raw bars *.

Nutrient	Recipe 1	Recipe 2	Recipe 3	Recipe 4
Protein (%)	12.13 ± 1.1	9.99 ± 0.8	9.72 ± 0.8	9.83 ± 0.7
Total lipids (%)	18.77 ± 1.1	19.37 ± 1.1	18.56 ± 1.2	18.26 ± 1.1
Total sugars (%)	51.11 ± 2.4	44.98 ± 2.2	45.40 ± 2.1	47.14 ± 2.2
Total fiber (%)	6.67 ± 0.3	11.86 ± 0.8	13.86 ± 0.9	6.43 ± 0.3
Calories (kcal)	453.68 ± 10.1	457.16 ± 9.7	461.11 ± 11.2	425.87 ± 10.4

* Mean values ± SD.

**Table 4:** Total phenolic content (TPC), Trolox equivalent antioxidant capacity (TEAC), and the levels of vitamins B1, B3, and B6 in nutritive raw bars formulations *.

Raw Bars	TPC mg GAE/kg	TEAC µg/g echiv. Trolox	B1 µg/g	B3 µg/g	B6 µg/g
Recipe 1	926.32 ± 18.15	2620 ± 23.64	5.83 ± 0.37	33.66 ± 5.47	19.22 ± 3.36
Recipe 2	749.1 ± 14.23	2010 ± 25.67	3.12 ± 0.17	19.08 ± 3.55	21.22 ± 4.07
Recipe 3	489.45 ± 8.49	1390 ± 15.67	2.66 ± 0.08	18.63 ± 3.12	19.67 ± 3.94
Recipe 4	844.23 ± 16.34	2390 ± 25.87	2.33 ± 0.07	21.31 ± 4.39	17.77 ± 3.11
*p*-value	2.0206 × 10^−9^	1.7693 × 10^−11^	1.3155 × 10^−7^	0.007365	0.5628

* Mean values ± SD

**Table 5:** Release of Phenolic Compounds and Vitamins B1, B3, and B6 from raw bars during Simulated Gastrointestinal Digestion *.

Simulated Fluid	TPC mg GAE/kg	TEAC µg/mL Trolox	B1 µg/g	B3 µg/g	B6 µg/g
R1 SSF	185.07 ± 10.87	129.45 ± 9.21	2.42 ± 0.14	15.59 ± 0.94	7.08 ± 0.67
R1 SGF	71.92 ± 4.47	61.27 ± 5.14	1.56 ± 0.09	9.86 ± 0.74	5.92 ± 0.54
R1 SIF	48.19 ± 2.37	49.68 ± 3.67	1.24 ± 0.07	8.14 ± 0.67	5.89 ± 0.55
R2 SSF	184.42 ± 11.68	138.77 ± 11.34	1.52 ± 0.08	6.45 ± 0.51	8.94 ± 0.71
R2 SGF	109.35 ± 8.67	81.74 ± 6.93	0.82 ± 0.06	5.83 ± 0.48	5.4 ± 0.48
R2 SIF	96.51 ± 8.07	75.71 ± 6.37	0.59 ± 0.02	5.74 ± 0.46	5.35 ± 0.48
R3 SSF	171.69 ± 8.99	116.09 ± 9.07	1.08 ± 0.08	7.9 ± 0.68	9.71 ± 0.82
R3 SGF	108.27 ± 7.85	95.75 ± 8.74	0.93 ± 0.09	5.24 ± 0.49	5.25 ± 0.41
R3 SIF	55.74 ± 4.71	42.54 ± 3.43	0.62 ± 0.05	5.1 ± 0.46	4.34 ± 0.39
R4 SSF	102.01 ± 8.67	100.61 ± 9.07	1.02 ± 0.09	7.75 ± 0.71	6.36 ± 0.61
R4 SGF	94.79 ± 9.13	89.27 ± 7.34	0.67 ± 0.04	6.12 ± 0.51	5.07 ± 0.47
R4 SIF	80.55 ± 7.59	80.696 ± 7.08	0.42 ± 0.02	6.27 ± 0.57	4.72 ± 0.39
*p*-value	9.4615 × 10^−7^	0.0001494	1.810 × 10^−5^	8.098 × 10^−14^	6.8123 × 10^−11^

* Mean values ± SD

**Table 6:** Recovery Rates of Total Phenols, Antioxidant Capacity, and Vitamins B1, B3, and B6 for Different Recipes after Simulated Digestion.

Recipe	Recovery (%)				
	Total Phenols	Antioxidant Capacity	B1	B3	B6
Recipe 1	34.2–36.2	8.7–10.4	87.4–90.3	99.2–99.8	96.1–99.2
Recipe 2	50.1–53.7	13.1–15.5	91.1–95.3	92.3–95.7	90.7–93.4
Recipe 3	67.4–69.4	16.9–19.4	97.4–98.9	96.1–98.9	97.4–99.3
Recipe 4	31.7–34.5	10.2–12.7	89.7–92.6	91.4–96.8	89.7–92.1

**Table 7:** Pearson Correlation Matrix: Linear Relationship between Pairs of Variables Analyzed in the Study.

	TPC mg GAE/L	TEAC µg/mL Echiv Trolox	B1	B3	B6
TPC mg GAE/L	1				
TEAC µg/mL echiv Trolox	0.950	1			
B1	0.566	0.493	1		
B3	0.423	0.356	0.884	1	
B6	0.804	0.734	0.521	0.334	1

## Data Availability

The data presented in this study are available on request from the corresponding author. The data are not publicly available due to privacy restrictions.
